# Ecophysiological Responses of Triterpene Glycosides in Buds of *Aralia elata* (Miq.) Seem. to Late Spring Frost with Soil-Mediated Effects

**DOI:** 10.3390/plants14142115

**Published:** 2025-07-09

**Authors:** Ning Wang, Dandan Zang, Wenbo Zhao, Yudong Sun, Wei Zhang, Yadong Duan

**Affiliations:** 1 State Key Laboratory of Black Soils Conservation and Utilization, Northeast Institute of Geography and Agroecology, Chinese Academy of Sciences, Harbin 150081, China; wangning@iga.ac.cn (N.W.); zangdandan@iga.ac.cn (D.Z.); 1696156617@163.com (Y.S.); 2 Huma Cold Temperature Plant Germplasm Resources Protection Field Scientific Observation and Research Station of Heilongjiang Province, Northeast Institute of Geography and Agroecology, Chinese Academy of Sciences, Huma 165100, China; 3 Institute of Rural Revitalization Science and Technology, Heilongjiang Academy of Agricultural Sciences, Harbin 150028, China; zwb08152020@163.com; 4 Yichun Branch of Heilongjiang Academy of Forestry, Yichun 153000, China; zw81025259@163.com

**Keywords:** false spring, late spring frost, *Aralia elata*, triterpene glycoside, Araloside, non-wood forest product

## Abstract

Late spring frost (LSF) poses a threat to temperate forest ecosystems; however, its combined effects with soil properties on triterpene glycosides in the buds of valuable shrubs are still unclear. In this study, natural *Aralia elata* (Miq.) Seem. populations were investigated in 15 counties in Heilongjiang and Jilin provinces in Northeast China. Buds were sampled in 3–5 cm length and used for determining triterpene glycosides (TGs) of Araloside VI, Araloside V, and 4-F8 (structural analogs) in spring of 2023. LSF in Heilongjiang showed longer days reaching 20 °C (CD20) (6.0 ± 2.5 d), LSF number (NLSF) (1.8 ± 0.5 times) and duration (DLSF) (21.5 ± 5.2 d), and days of temperature rise (DTR) (15.9 ± 3.8 d) compared to Jilin (4.4 ± 0.4 d, 1.2 ± 0.4 times, 17.4 ± 3.9 d, 12.0 ± 3.3 d, respectively). Araloside VI (0.30–0.59%) was positively driven by DLSF but negatively driven by DTR. Araloside V (0.04–0.17%) and 4-F8 (0.09–0.44%) were positively influenced by the lowest temperature, DTR, and CD20, negatively influenced by NLSF, and slightly influenced by organic matter. In LSF-prone regions, soil organic matter and nutrient availability do not need to be enriched, and soil pH should be higher than 5.7 if high TGs are the objective in *A. elata* buds.

## 1. Introduction

Late spring frost (LSF), also termed as “false spring,” poses a significant threat to temperate forest ecosystems [1,2,3,4]. It can disrupt critical phenological processes during the vulnerable spring season, when LSF occurs with abnormal warm spells and sudden temperature drops that, together, trigger premature bud burst or leaf-out [5,6]. This phenomenon is becoming increasingly frequent due to climate warming, which advances the spring phenology and increases the risk of frost exposure [7,8]. LSF can damage emerging tissues by reducing photosynthetic productivity and delaying phenological events [9,10]. Researchers have paid attention to LSF for over 60 years [5,8], but the mechanism of botanical responses to this extreme weather event remains uncertain.

Forests are complex ecosystems that provide essential services for carbon sequestration, nutrient cycling, and habitats for diverse flora and fauna [11,12]. Highly valued understory plants are also defined as non-wood forest products (NWFPs) for their medicinal, edible, or cultural uses [13,14]. Commonly seen NWFPs include edible fruits, medicinal herbs, and other understory plants, which are vital for both ecological and economic sustainability, particularly in temperate and boreal forests [14,15]. However, these ecosystems face increasing threats from climate-driven phenomena, and LSF stands at the head of the queue of climatic threat due to its severe impacts on plant phenology and productivity for NWFPs [16,17]. As climate models predict warmer winters with reduced snow cover, understanding these dynamics is becoming increasingly urgent to inform conservation and management strategies for NWFPs [18,19]. Spring is critical for bud burst and growth in many NWFP species; however, LSF may impair bud growth and quality by disrupting the synthesis and accumulation of bioactive compounds with remarkable ecological and economic value [20,21]. Despite the recognized effects on plant phenology, evidence regarding the effects of LSF on the NWFP bud quality remains limited, necessitating further research to inform conservation and sustainable management strategies.

The vulnerability of understory plants to LSF is modulated by various biotic and abiotic factors, with soil properties emerging as critical covariates that influence plant resilience and bud quality [22,23]. The susceptibility of boreal and temperate forest ecosystems to climate change underscores the need to understand how soil properties and LSF interact to affect NWFP viability [24]. Soil properties significantly influence the growth and survival of NWFP understory plants, mediating the effects of LSF on bud quality [25,26]. The soil nutrient availability governs the nutrient uptake and storage, which are crucial for bud development during the subsequent growing season [27,28]. For example, soil freezing can delay the root activity and limit the nutrient uptake, which are critical for bud development [29]. Higher nitrogen (N) and phosphorus (P) availability in the soil can enhance foliar nutrient reserves, supporting robust bud formation [15,30]. The soil pH is another critical factor that can limit plant responses to frost by altering nutrient accessibility, with acidic soils potentially exacerbating stress under LSF conditions [22,31]. Additionally, the soil organic matter regulates the nutrient supply and soil aeration, both of which influence spring growth and frost resistance by improving the root function and water retention [32,33,34]. Soil electrical conductivity (EC) may also pose a threat, as high EC levels can exacerbate frost-induced damage by increasing the physiological stress [18]. Studies have shown that altered soil conditions can influence the nutrient dynamics and plant phenology, potentially amplifying the effects of LSF [35,36]. Despite these insights, the combined effects of LSF and soil properties on the bud quality remain poorly understood [37,38], necessitating further research to elucidate their interactions.

Triterpenes account for the edible and medicinal qualities of the buds of many NWFP species [39,40,41]. Triterpenes are a class of organic compounds (general formula C30H48) derived from six isoprene units, resulting in a 30-carbon skeleton. The synthesis of triterpenes is involved in the balance between carbon and nutrient assimilation, which is highly relevant to rhizospheric soil properties. This hypothesis suggests that plants allocate resources between growth (primary metabolism) and defense/secondary metabolism, based on the availability of carbon and nutrients [42]. In conditions where carbon (C) is abundant (e.g., high light) but soil nutrients are scarce, plants may allocate more resources to carbon-based secondary metabolites, including the synthesis of triterpenes. Conversely, if the soil nutrients are abundant but carbon is limited (e.g., low light), plants might prioritize growth over secondary metabolite production. Under the first condition, high soil nitrogen (N) availability can form an optimum N level that provides C precursors and energy for triterpene synthesis [43]. In addition, enriched reserves of phosphorus (P) and potassium (K) in soils are vital for overall plant health and energy metabolism, indirectly supporting the biosynthetic pathways of triterpenes [44]. Under the second condition, improper soil properties trigger a defensive mechanism that enhances secondary production, including triterpene synthesis. These stressful soil conditions include nutrient deficiency [45], extreme pH value [46], microbial signals [47], pathogen and pest pressure [48], drought or waterlogging [49], and poor physical structure [50]. These pieces of knowledge have rarely been re-examined for their interactive effects on triterpene synthesis in NWFP plants subjected to extreme weather events.

*Aralia elata* (Miq.) Seem. is a perennial woody plant belonging to the Araliaceae family. It is a significant NWFP species widely distributed in Northeast China, Japan, and Korea, where it is highly valued for its medicinal and edible properties [51,52]. As an NWFP, *A. elata* plays a critical role in forest biodiversity and local economies, particularly in less-developed regions, where it supports subsistence and trade [53]. Its newly growing buds are consumed as wild vegetables, prized for their unique flavor and nutritional content, including polysaccharides, flavonoids, and triterpene saponins [54,55]. Buds of *A. elata* are highly valuable because of their enriched triterpene glycosides, such as Araloside V and VI [39]. These bioactive compounds contribute to their pharmacological effects and are staples in traditional medicine and functional foods [56,57]. In Japan, its buds are popular wild vegetables, often prepared as salads or pickles [54]. Bud growth coincides with periods of low soil fertility, potentially affecting quality, although evidence remains limited [58]. However, overexploitation and environmental changes threaten natural populations and habitat suitability under the stress caused by climatic threats [59,60]. Soil properties may also interact with LSF to influence the triterpene glycoside content, necessitating studies to evaluate these effects under varied conditions [31,58]. Sustainable in-forest cultivation practices are essential for mitigating these challenges, and more evidence is needed to maintain or increase secondary metabolite production in LSF.

In this study, 15 counties in Northeast China with natural *A. elata* populations were selected as the study area. The objectives of this study were to (1) determine the weather attributes of LSF in stands of *A. elata* populations and (2) detect the combined effects of LSF and soil properties on typical triterpene glycosides in *A. elata* buds. We also aimed to provide a theoretical reference for policymaking for the protocol of cultivating *A. elata* resources with a high bud quality in an era exposed to LSF.

## 2. Results

### 2.1. Growth and Triterpene Glycosides in A. elata Buds

#### 2.1.1. Growth of *A. elata* Buds

**Figure 1:**
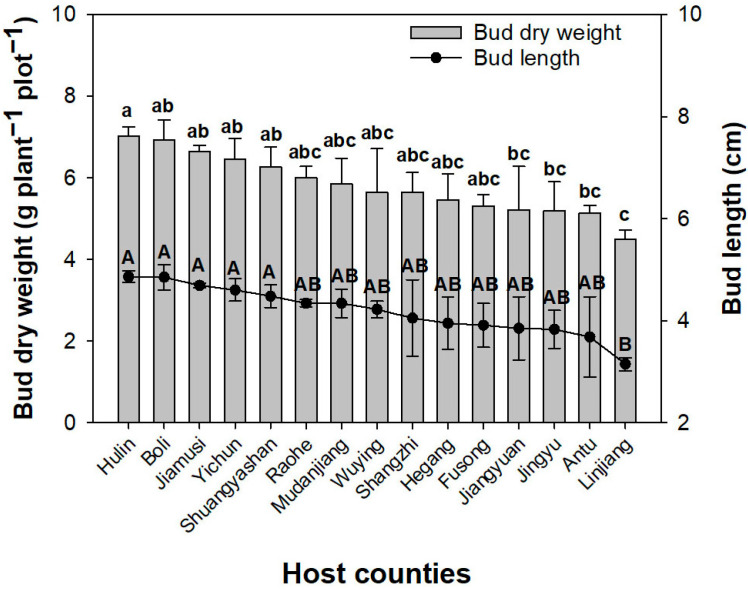
Variations in the dry weight and length of *Aralia elata* (Miq.) Seem. buds were observed in natural population plots located in different host counties. Error bars indicate standard error. Different letters indicate significant differences among the counties. Lower case letters a, b, and c indicate differences in bud dry weight; capital letters A and B indicate differences in bud length.

The bud dry weight was lower in Linjiang County of Jilin Province than in the Hulin, Boli, Jiamusi, and Yichun counties in Heilongjiang Province (Figure 1). The bud dry weight in Hulin County was greater than that in Jiangyuan, Jingyu, and Antu counties in the Jilin Province. The bud length was shorter in the Linjiang County of Jilin Province than in the Hulin, Boli, Jiamusi, Yichun, and Shuangyashan counties of Heilongjiang Province.

#### 2.1.2. Triterpene Glycosides in *A*. *elata* Buds

**Figure 2:**
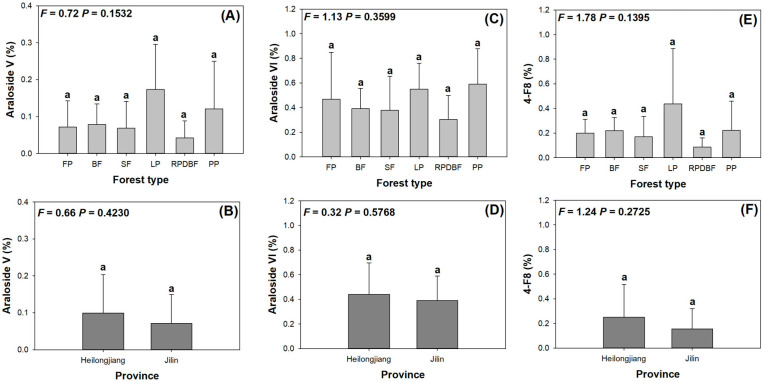
Variations in triterpene glycosides (Araloside V, **A**,**B**; Araloside VI, **C**,**D**; 4-F8, **E**,**F**) in *A. elata* buds were distributed in plots subjected to different forest types (**A**,**C**,**E**) and located in two provinces (**B**,**D**,**F**). Error bars indicate the standard error. Different letters indicate significant differences among counties. Forest type abbreviations: SF, second forest; PP, pine plantation; LP, larch plantation; RPDBF, red pine (*Pinus koraiensis*) and deciduous broadleaf mixed forest; BF, birch forest; FP, fir plantation. The results were assigned as the percentile rate of the triterpene amount relative to the dry weight mass in buds.

As shown in Figure 2, none of triterpene glycosides (TGs) showed significant responses to either varied forest types or provincial variations (Figure 2). Araloside V, Araloside VI, and 4-F8 ranged from 0.04 to 0.17% (Figure 2A,B), 0.30–0.59% (Figure 2C,D), and 0.09–0.44% (Figure 2E,F), respectively. Therefore, the amount of Araloside VI was higher than those of Araloside V and 4-F8.

**Figure 3:**
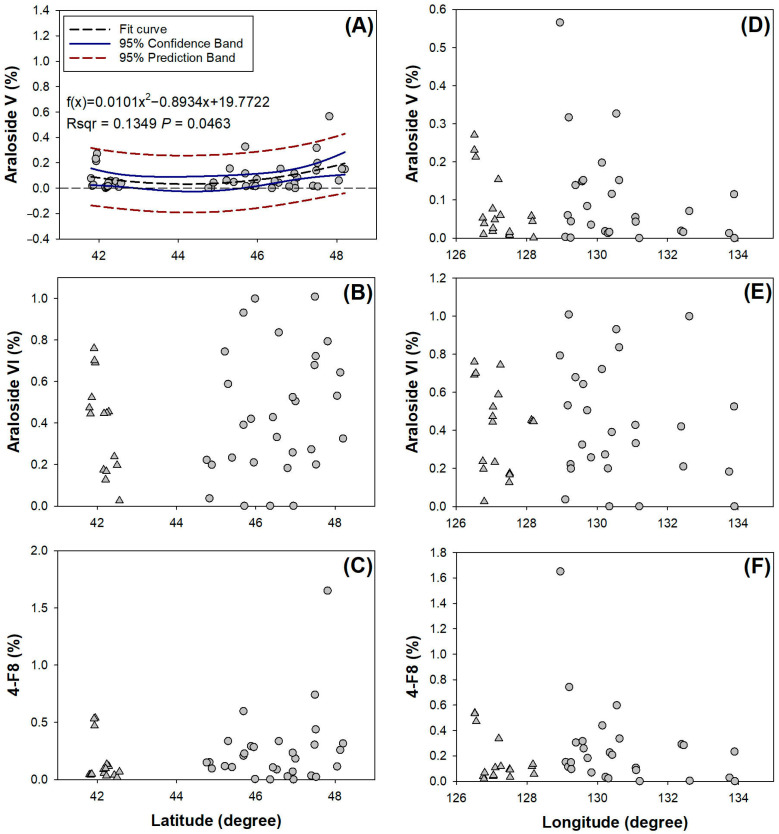
Geographical responses of Araloside V (**A**,**D**), Araloside VI (**B**,**E**), and 4–F8 (**C**,**F**) to latitudinal (**A**–**C**) and longitudinal gradients (**D**–**F**) of host plots. In (**A**), the dashed line labels the zero level along the latitudinal gradient, and none of the parts of the 95% confidence band and 95% prediction band below the dashed line have essential meanings. Round dots mark observations that can be fitted by curves across the whole spectrum of topographical gradients or parts of observations distributed in Heilongjiang Province (over 44° N), in plots over 128.5° E, and trigonal dot mark observations in Jilin Province (<44° N) or in plots distributed below 128.5° E. Results are assigned as the percentile rate of triterpene amount relative to the dry weight mass in buds.

The responses of Araloside V observations to latitudinal gradient were fitted using a quadratic polynomial function (Figure 3A). At 44.23° N, it was theoretically indicated to have the lowest level of Araloside V (0.16‰). The amount of Araloside VI in the buds of *A. elata* in plots within the latitudinal range of Jilin Province showed negative responses to latitude, with a slope of 0.6758 (Figure 3B). The responses of 4-F8 to the latitudinal gradient were divided in the range of approximately 43.0°–44.5° N, with 4-F8 values near 48° N increasing up to be higher than 1.5% (Figure 3C). Along the longitudinal gradient, no observations could be fitted by any curve (Figure 3D–F). Distributions of Araloside V and 4-F8 showed occ Authors make sure all gene symbols and species’ names be written in italics throughout the paper, and proteins and enzymes were not be written in italics. These have been checked throughout the manuscript including those in figures. asionally high records in plots at longitudes of approximately 128° E (Figure 3D,F).

### 2.2. Soil Properties in A. elata Plots

#### 2.2.1. Variations in Soil Properties Among *A. elata* Plots

**Figure 4:**
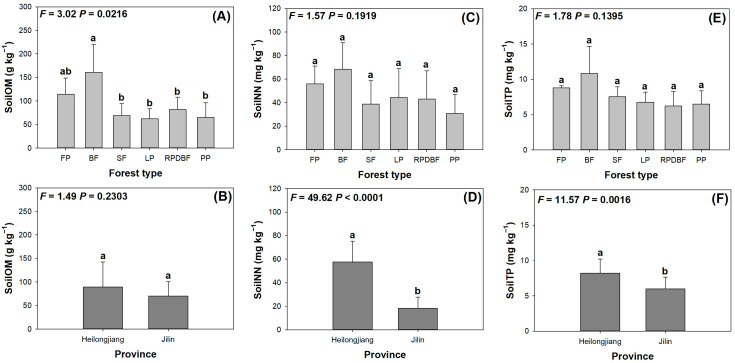
Variations in soil properties (soil organic matter, SoilOM, **A**,**B**; soil nitrite N content, SoilNN, **C**,**D**; soil total P content, SoilTP, **E**,**F**) in plots of *A. elata* populations distributed subjected to different forest types (**A**,**C**,**E**) and located in two different provinces (**B**,**D**,**F**). Error bars indicate the standard error. Different letters indicate significant differences among counties.

Among all variables reflecting soil properties, only soil organic matter (SoilOM), soil nitrite N content (SoilNN), and soil total P content (SoilTP) showed significant responses (Figure 4). SoilOM was higher in the BF type than in other types, except for FP (Figure 4A), but it did not show a significant difference between provinces (Figure 4B). SoilNN also failed to vary among forest types (Figure 4C), but it was 217.7% higher in Heilongjiang Province than in Jilin Province (Figure 4D). Again, SoilTP did not respond to forest-type variation (Figure 4E) but was higher in Heilongjiang (37.1%) than in Jilin (Figure 4F). In addition, soil total N content (SoilTN) ranged in 0.26–0.53 g kg ^−1^ (*F* = 0.86, *p* = 0.5336), soil ammonium N content (SoilAN) ranged in 40.13–58.44 mg kg ^−1^ (*F* = 1.18, *p* = 0.3373), soil available P content (SoilAP) ranged in 75.00–152.42 mg kg ^−1^ (*F* = 1.58, *p* = 0.1810), soil pH value (SoilpH) ranged in 5.21–5.73 (*F* = 2.01, *p* = 0.0890), and soil electrical conductance (SoilEC) ranged in 15.47–27.55 µS cm ^−1^ (*F* = 0.92, *p* = 0.4892).

#### 2.2.2. Inner Relationships Between Paired Variables About Soil Properties

**Figure 5:**
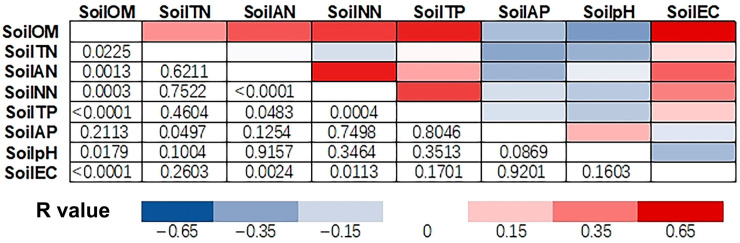
Correlation heatmap of relationships between paired variables among soil properties in plots of national *A. elata* populations. Cells in different colors present *R* values in accordance with the *p* values mirrored in symmetry.

The SoilOM was positively correlated with SoilTN, SoilAN, SoilNN, SoilTP, and SoilEC and negatively correlated with the soil pH (Figure 5). The SoilAP also showed a negative relationship with SoilTN, and the SoilEC showed a positive relationship with SoilAN and SoilNN.

### 2.3. LSF Attributes

#### 2.3.1. Variations in LSF Attributes

The forest type did not have any effect on the LSF attributes, but the provincial variation showed a significant impact on most of variables (Table 1). The LSF in Heilongjiang showed higher CD0, CD20, NLSF, DLSF, and DTR than that in Jilin. The TDR was also higher in Heilongjiang, whereas the HTLSF was higher in Jilin.

#### 2.3.2. Inner Relationships Between Paired Variables About LSF Attributes

**Figure 6:**
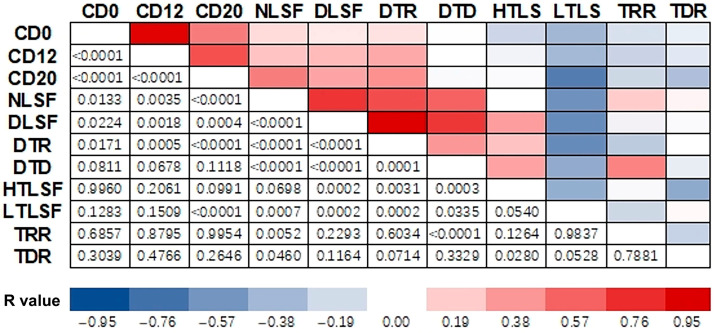
Correlation heatmap of relationships between paired variables among LSF attributes in plots of national *A. elata* populations. Cells in different colors present *R* values in accordance with the *p* values mirrored in symmetry.

As shown in Figure 6, strong positive correlations (*R* > 0.5 in deep red) were observed between CD12 and CD20, NLSF and DLSF, and TRR and DTD. Strong negative correlations (*R* < −0.5, moderate to deep blue) were found between CD0 and CD12 and between CD0 and CD20. Negative correlations were also found between pairs of the NLSF-DTR and NLSF-DTD.

### 2.4. Driving Forces of Soil and LSF on Triterpene Glycosides in A. elata Buds

**Figure 7:**
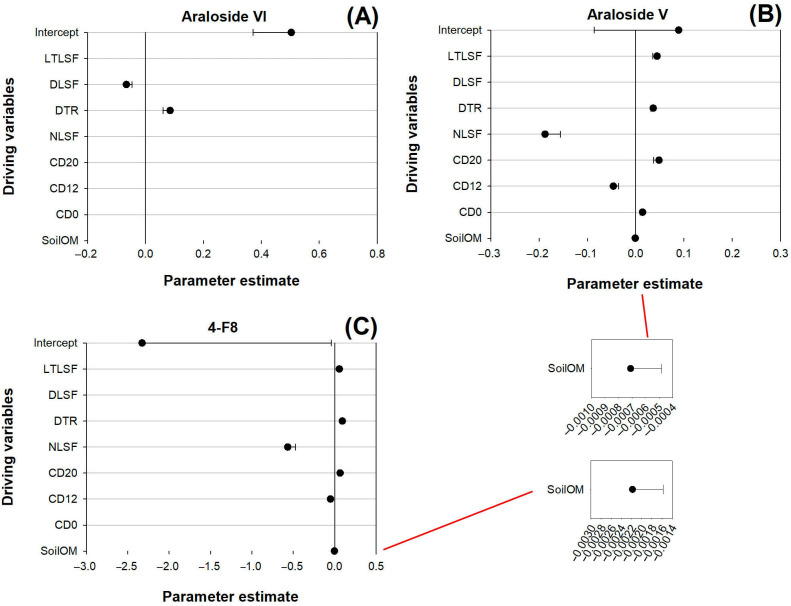
Multivariate linear regressions of Araloside VI (**A**), Araloside V (**B**), and 4-F8 (**C**) in *A. elata* buds against variables reflecting LSF (LTLSF, DLSF, DTR, NLSF, CD20, CD12, and CD0) and soil organic matter content (SoilOM). Error bars indicate the zero-line standard errors of the parameter estimates. All dots represent significant contributions, passing the level of 0.05.

The regression of Araloside VI was positively driven by DLSF and negatively driven by DTR (Figure 7A). Araloside V regressed under positive forces from LTLSF, DTR, CD20, and CD0, while it was also negatively driven by NLSF and CD12, with a tiny negative force from SoilOM (Figure 7B). Similarly, 4-F8 was positively driven by LTLSF, DTR, and CD20, and received negative driving forces from NLSF, CD12, and SoilOM (Figure 7C).

## 3. Discussion

### 3.1. LSFs in Counties Containing A. elata Plots: What Deserves Attention?

Our study employed a robust methodology to characterize LSFs in counties hosting *A. elata* plots by leveraging high-resolution NOAA GHCN temperature data to define LSF events based on warm spells followed by subzero temperature drops. This approach ensures the precise identification of LSF events by capturing subtle variations in frost attributes, such as duration (DLSF), temperature decline rate (TDR), and cumulative warm days prior to frost. The use of daily temperature records enabled us to detect LSF events with high temporal accuracy, as demonstrated by our ability to pinpoint frost occurrences within a narrow phenological window that is critical for *A. elata* bud development [6,8]. Additionally, integrating plot-specific microclimate data allows for the consideration of local topographic influences and enhances the spatial precision of LSF assessments compared with broader regional models [20]. These methodological strengths facilitated a nuanced evaluation of the impact of LSF on *A. elata*, distinguishing our study from generalized forest studies.

The LSF characteristics varied significantly between Heilongjiang and Jilin provinces. Heilongjiang exhibited more frequent and severe LSF events with higher DLSF and TDR, likely because of its colder continental climate and greater temperature fluctuations. Jilin showed milder springs and experienced fewer frost days; hence, local LSFs were characterized by less abrupt temperature declines. This aligns with the regional trends of decreasing frost risk in warmer temperate zones [61]. Compared to prior studies, our LSF events were marked by prolonged warm spells before frosts, a pattern less emphasized in European or North American forests, where shorter warm periods precede frosts [5,7]. Crowded populations and dense industrial activities may account for this difference, but more confirmatory evidence is needed. The prolonged warming in our study heightened the *A. elata* bud vulnerability as the advanced phenology increased the frost exposure [9].

The uniqueness of the impact of LSF on *A. elata* lies in its specific phenological sensitivity compared with that of general forest species. Unlike deciduous trees (such as *Fagus sylvatica*), which may refoliate post-frost [62], *A. elata* buds rarely recover from TGs affected by frost-induced metabolic disruption. Key LSF attributes included TDR, which exhibited a strong negative correlation with the TG content. This underscores the critical role of frost severity in biochemical damage [63]. Additionally, DLSF was positively correlated with bud biomass reduction, which was less pronounced in studies of coniferous species with greater frost tolerance [64]. Together, these findings highlight the distinct ecological niche of *A. elata* and suggest that LSF management strategies for NWFPs require tailored approaches, accounting for species-specific frost responses and regional climatic variability [65].

### 3.2. Soil Properties in A. elata Plots: Do They Matter?

It was revealed that the soil organic matter (SOM) had a slight negative effect on Araloside V (0.04–0.17%) and 4-F8 (0.09–0.44%) concentrations. This suggests that excessive SOM may disrupt nutrient dynamics and potentially limit TG synthesis due to the immobilization of available N resources [66] and possibly anaerobic conditions [67]. Conversely, Araloside VI (0.30–0.59%) was not significantly affected by SOM, indicating a different response mechanism from that of Araloside V and 4-F8. We do not have specific evidence to explain this phenomenon, but this may be caused by the different derivatives of the same triterpenoid scaffold differing in their degree of saturation and functional group arrangement in the five-membered ring. The soil pH ranged from 5.21 to 5.73 and was negatively correlated with SOM with a pH above 5.7 recommended for higher TG yields. This can be explained by the C-N balance theory that the pH 5.7 nearly reached the highest upper limit (5.73), which may have stressed *A. elata* and benefited the TG synthesis. The soil nutrient availability can be determined by the SoilTN and TP concentrations, which are not critical for TG enrichment. It is likely that the soil T and P availability was sufficient for *A. elata* growth, and nutrient sufficiency made nutrients irrelevant to the effects on TG. A higher SOM in birch forests enhances nutrient retention but impairs TG production, likely due to altered nutrient dynamics. Taking together, these findings suggest that the dual facets of moderate SOM content and higher pH are crucial for optimizing TG concentrations in *A. elata* buds.

### 3.3. Growth and TGs in Buds of A. elata: Response and Trend

The bud growth in *A. elata* was measured by the bud dry-weight and bud length, both of which exhibited a significant variation across counties, reflecting environmental influences on plant vigor. The bud dry weight was higher in the northern counties, likely because of better acclimation to the local environment. However, the bud length showed less consistent patterns, with longer buds in counties with higher precipitation levels. This suggests that moisture availability is a key factor that determines bud elongation. These growth metrics underlie the observed TG trends. Higher TG concentrations were recorded in northern and western counties, where lower temperatures and reduced solar radiation favor secondary metabolite synthesis.

Compared to other studies, Araloside V and Araloside VI levels in our *A. elata* buds ranged between 1.2–2.5 mg g ^−1^ and 0.8–1.9 mg g ^−1^, respectively. These align with reported ranges for related species, though that in 4-F8 (0.3–0.7 mg g ^−1^) was lower than in high-altitude populations [63]. The latitudinal gradient decreases the Araloside V content in southward plots, which reflects temperature-driven metabolic shifts, as warmer climates suppress the glycoside production. The longitudinal gradient did not lead to significant gradient responses in TG levels, which suggests that longitudinal gradients have irrelevant impacts on biosynthetic pathways. These location-dependent trends suggest that TG accumulation is more likely to occur in cooler, nutrient-rich environments. Management strategies for *A. elata* cultivation should prioritize northern and western counties to optimize TG yields while accounting for climatic and edaphic factors influencing the bud growth and metabolite profiles.

These location-dependent trends suggest that TG accumulation in *A. elata* buds is more likely to occur in cooler, nutrient-rich environments. To enrich policymaking, cultivation protocols should integrate high-resolution climate data to target sites with optimal LSF attributes, ensuring that bud growth aligns with periods of high precipitation to support elongation, such as observations in counties of Hulin and Yichun. Soil management should maintain a pH above 5.7 and avoid excessive SOM to avoid negative effects on TG content. Policies should promote sustainable harvesting to prevent overexploitation, preserve bud dry weight and length variation, and support regional conservation efforts for long-term ecological and economic benefits.

### 3.4. Combined Effects of LSF and Soil Properties on TGs in A. elata Buds

The interplay between LSF and soil properties significantly influenced the TG content in *A. elata* buds, revealing complex ecological dynamics that shaped secondary metabolite production. The day length of LSF positively drove Araloside VI accumulation, suggesting that prolonged frost exposure enhanced specific biosynthetic pathways, possibly due to stress-induced metabolic shifts [68]. Conversely, Araloside V and 4-F8 were positively influenced by the lowest temperature during frost (LTLSF) and cumulative warm days (CD20), indicating that temperature extremes and warm spells prior to frost events stimulated TG production. These findings align with prior research showing that temperature fluctuations during spring phenology can alter secondary metabolite profiles in understory plants [69].

The soil properties further mediate these effects by influencing nutrient availability, which is critical for TG synthesis. The SOM exerted a slight negative effect on Araloside V and 4-F8 contents, potentially due to excessive organic matter altering nutrient dynamics under frost conditions [25]. Regarding the positive relationship between SOM and nutrient availability, enriched soil nutrients were not required to accumulate TGs in A. elata buds subjected to LSF. The negative relationship between the SOM and pH suggests that the soil pH values (5.21–5.73) are too low for bud resistance to LSF. Higher SOM levels were observed in birch forests, where they correlated with increased nutrient retention but impaired TG accumulation.

### 3.5. Limits of This Study

First, the temporal scope of the bud and soil sampling was confined to a single growing season, potentially overlooking the inter-annual variability in TG production and soil properties influenced by cross-year climatic fluctuations. The multiyear data revealed advanced warm spells and strengthened the robustness of our conclusions. Second, this study focused on a limited set of soil parameters (soil organic matter, total nitrogen, and total phosphorus), which may not fully represent the complex soil chemistry affecting TG synthesis. Incorporation of micronutrients or soil microbial dynamics may reveal additional drivers. Third, LSF was monitored and characterized on a regional scale. This possibly masked fine-scale variations within forest patches, which influenced the microclimate and TG accumulation. Finally, this study did not account for genetic diversity among *A. elata* populations, which can significantly affect the TG profiles and growth responses to environmental gradients. Addressing these limitations in future studies will enhance our understanding of the ecological and biochemical dynamics of *A. elata*.

### 3.6. Conclusions

This study revealed that LSF and soil organic matter synergistically influenced the TG content in *A. elata* buds. Higher SOM levels coupled with the prolonged LSF duration enhanced Araloside VI production, whereas extreme frost temperatures and warm spell days boosted Araloside V and 4-F8 accumulation. The small negative contribution of the SOM to TGs predicted the low nutrient availability and the requirement for a higher pH as conditions for promoting TG biosynthesis under LSF stress. Overall, to effectively cultivate *A. elata* in LSF-prone regions, the soil organic matter and nutrient availability were not required to fall to enriched levels, and temperatures over 12 °C should be considered to avoid unpredictable declines in bud TGs.

Three major driving forces emerged from these interactions. First, a prolonged late spring frost (LSF) duration (DLSF, 21.5 ± 5.2 days in Heilongjiang) triggered stress responses in *A*. *elata,* which enhanced the Araloside VI accumulation (0.30–0.59%) by inducing metabolic shifts that favored glycoside biosynthesis. Second, temperature extremes, including the lowest temperature during LSF (LTLSF, −5.5 ± 2.0 °C) and cumulative warm days above 20 °C (CD20, 6.0 ± 2.5 days), together stimulated the Araloside V (0.04–0.17%) and 4-F8 (0.09–0.44%) production. These findings reflect the heightened metabolic activity under thermal stress. Third, the soil nutrient availability variables of the SoilNN (217.7% higher in Heilongjiang) and SoilTP (37.1% higher in Heilongjiang) both supported metabolic resilience, which enabled robust bud development despite LSF stress. These findings highlight the need for integrated management strategies to optimize *A. elata* cultivation under changing climatic conditions. Policymakers should prioritize northern regions, such as Hulin and Yichun, with enlarged areas of *elata* populations with meteorological condition evaluation using NOAA GHCN data to identify optimal LSF-prone sites. Maintaining a soil pH above 5.7 and avoiding excessive soil organic matter should be implemented in practice to enhance TG yields. Sustainable harvesting and conservation policies are essential to protect natural populations and ensure the long-term ecological and economic sustainability of this non-wood forest product.

## 4. Materials and Methods

### 4.1. Study Area and Plot Target

This study was conducted by investigating natural *A. elata* populations in plots located in the counties of Heilongjiang and Jilin provinces in Northeast China [55]. The initial targets of these plots were designated according to previous publications [15,52,59,60]. Specific locations of populations were checked in July 2022, and locations may be adjusted to meet the requirements, if necessary. Our objectives were to identify natural *A. elata* populations that were not subjected to a visible anthropogenic impairment to any of the organs in the leaves, twigs, stems, or trunks. This was necessary because *A. elata* individuals usually suffer destruction, owing to illegal bud collection [58]. The final establishment of the plots and stand information are listed in Table 2.

### 4.2. Field Investigation and Sampling

Buds of *A. elata* were sampled as experimental materials from natural populations, and their inner triterpene glycosides (TGs) were taken as objective secondary metabolites in the chemical determination and as wild vegetable components in the spring of 2023. The unregulated harvest of the buds was long initiated by wild vegetable merchants, and the *A. elata* buds were mainly used for export to Japan, where they are widely consumed as a foodstuff at a remarkable price [70]. Therefore, most buds were picked when they grew to a length of 3–5 cm, and this height range was used as the minimum level for establishing the sampling date. That is, when the minimum bud length of *A. elata* individuals grew to at least 3–5 cm plant ^−1^ per plot, they could be prepared for sampling. All plots were monitored twice a week to check the growth status of the *A. elata* individuals until the bud growth met the sampling standard. During sampling, the entire bud was excised from host stems or twists. Two buds were collected from each plant, and four plants were harvested per plot. The excised bud was immediately measured for the length; then, the buds were reserved in nylon bags and transported to the laboratory on ice (0–4 °C) to prevent rot.

### 4.3. Air Temperature Records and Late Spring Frost Characterization

The air temperature was obtained from the open-access file transfer protocol (FTP) service hosted by the National Climatic Data Center (NCDC) charged by the National Oceanic and Atmospheric Administration (NOAA) [71]. The air temperature data were recorded by the Global Historical Climatology Network (GHCN) across a chronological range from 1942 to the most recent year of interest. Data were recorded every 3 h on a daily basis, which facilitated obtaining the highest and lowest air temperature records and their averages. Geographies of air temperature data targeted according to locations in the U.S. Air Force Stations (USAFS), which were matched by their closeness to our plots, have their USAFS codes shown in Table 3. The daily air temperatures were recorded continuously from the start of 2023 until the day the buds were sampled. The termination of temperature monitoring depended on bud growth, which had to fall to a height of 3–5 cm. Hence, the ending-up dates varied in the plots at different locations because of variations in temperature-driven bud elongation. The sampling dates are listed in Table 3.

Late spring frost (LSF) was characterized by recognizing fluctuations in dynamic changes in air temperatures according to the following attributes:

(1) An LSF is formed during a false spring event with a warm spell, followed by a sudden temperature decline that can cause frost damage [6,68,72].
(2) The early stage warm spell lasts for at least five days, which enables accumulative temperature to induce bud burst and break dormancy [2,73]. The breakdown of dormancy is a necessary precondition that causes frost to occur with an essential probability.
(3) In the warm spell at the early stage, the air temperature rises above 12 °C, when bud burst can probably be induced and dormancy can be broken [74,75], and a higher temperature of 20 °C can increase the probability of these events [6,68,75].
(4) In a sudden decrease in the daily temperature, it must be lower than the dew point that reaches the freezing point, causing freezing damage [76,77].

**Figure 8:**
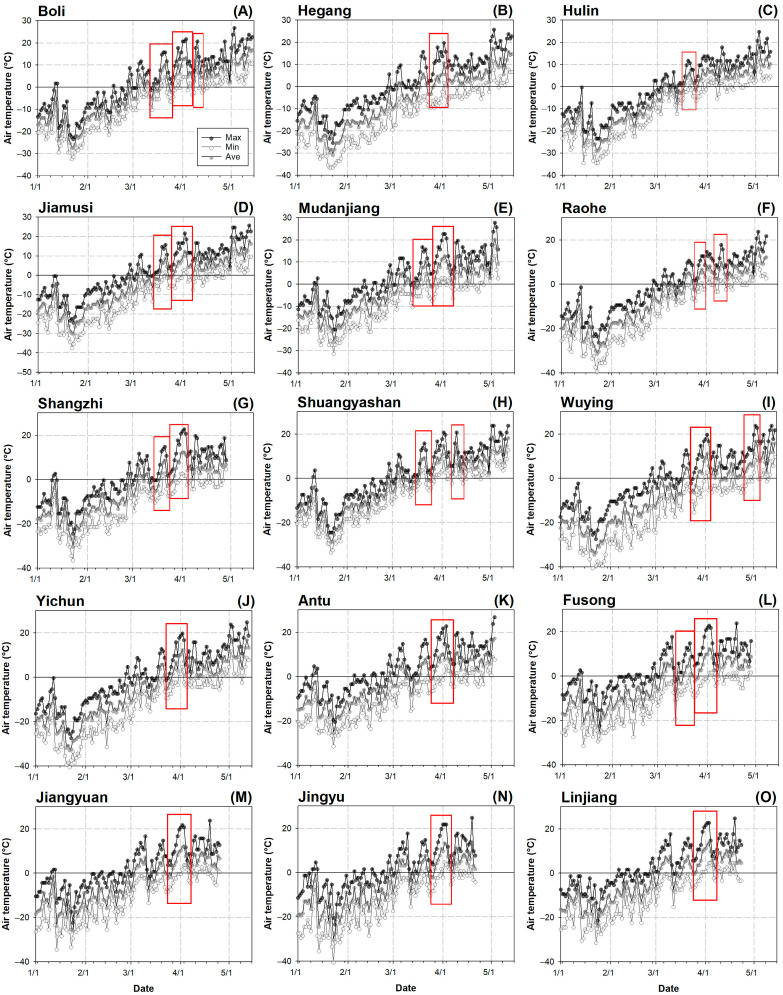
Dynamic changes in air temperature fluctuations of 2023 spring in plots located in host counties labeled in cells from A to O, including those in Heilongjiang province (**A**–**J**) and Jilin province (**K**–**O**). Red frames indicate periods of weather episodes that have typical LSF characteristics.

Based on these conditions, the spring temperature dynamics were analyzed, and typical LSF weather events are shown in Figure 8.

### 4.4. Field Investigation and Soil Properties

Field investigations were conducted at the same time that the plots were targeted, in July 2022. Each *A. elata* population was investigated in three replicate plots, and adjacent pairs of plots were separated from each other over a distance of 3 km. Each plot was set in a 900 m^2^ area in a 30 m × 30 m spacing arrangement, where trees were measured for species composition, height, diameter at breast height (DBH), stem density, and canopy density. Host forests were dominated by six types, including second forest (SF), pine plantation (mainly *Pinus sylvestris* var. *mongolica* Litv.) (PP), larch plantation (LP) (*Larix gmelinii* (Rupr.) Kuzen. or *L. olgensis* Henry), red pine (*P. koraiensis* Siebold et Zuccarini) and deciduous broadleaf mixed forest, birch (*Betula platyphylla* var. *mandshurica*) forest (BF), and fir (*Abies nephrolepis* [Trautv. ex Maxim.] Maxim.) plantation (FP). The forest structure was characterized as a tree height of 9.55 ± 1.50m (mean ± standard deviation, the same below), a DBH of 15.42 ± 5.49 cm, a stem density of 1412.32 ± 913.20 individuals, and a canopy density of 52.53 ± 20.33%.

The soil was collected from nine randomly placed subplots in the stand, each with an area of 9 m^2^ (3 m × 3 m). The forest floor was cleaned to ensure complete exposure to the soil surface. Two soil cores (inner diameter 10 cm) were collected at a depth of 20 cm from each subplot. Residues comprising wood chips, soil animal bodies, and dead plant tissues were removed from fresh soil samples. Two cores of soil were mixed for the host subplot, and all subplot soils were further mixed for the host plot. Fresh soil samples were screened through a 2 mm sieve and stored in nylon bags for transportation to the laboratory. Soil samples were air-dried at an indoor temperature until the dried weight was constant, and the samples were prepared for further determination.

Soil properties were characterized by organic matter (SoilOM), total nitrogen (N) content (SoilTN), ammonium N content (SoilAN), nitrate N content (SoilNN), total phosphorus (P) content (SoilTP), available P content (SoilAP), pH value (soil pH), and electrical conductance (SoilEC). The methodologies for determining these variables were adapted from previous studies of non-woody forest products [58,78,79]. The Walkley–Black method was used to determine SoilOM content, which involved oxidizing organic carbon with potassium dichromate (K_2_Cr_2_O_3_) in sulfuric acid (H_2_SO_3_), followed by titration with ferrous sulfate (FeSO_4_) to measure the remaining dichromate. Organic carbon is then converted to organic matter [80]. The soil TN was measured using an elemental analyzer (Vario MACRO cube, Elementar-Branch, Shanghai, mainland China), which received soil samples that were combusted at high temperatures. The resulting N oxides were reduced to gas, which was quantified using thermal conductivity detection [78,81]. Both SoilAN and SoilNN were extracted using 2 M potassium chloride (KCl) and analyzed using a flow injection system (Lachat Instru., Hach Ltd., Loveland, CO, USA). This method employs the indophenol blue reaction, in which ammonium reacts with hypochlorite and salicylate under alkaline conditions to produce a blue complex that can be measured at 660 nm [78,82]. The SoilNN was reduced to nitrite via cadmium reduction, followed by diazotization with sulfanilamide and coupling with N- (1-naphthyl) ethylenediamine to form a pink azo dye, as measured at 520 nm [78]. The soil TP was determined using inductively coupled plasma spectroscopy (Thermo ICAP-6000, ThermoFisher, Shanghai, China) on soil samples that had been digested with a mixture of sulfuric acid (H_2_SO_3_) and hydrogen peroxide (H_2_O_2_) to release phosphorus [78,83]. The SoilAP was extracted with distilled water and analyzed using the molybdenum blue method at 880 nm [78,84]. A soil-water suspension (1:2.5 ratio) was prepared, and the soil pH was measured using a 3020 pH detector (Jenway, Dunmow, UK) [58]. The soil EC was measured in microsiemens per centimeter (µS cm ^−1^) with a Leici DDSJ-308A conductivity meter with soil–water extract (1:5 ratio) [78].

### 4.5. Triterpene Glycoside Determination in Buds

**Figure 9:**
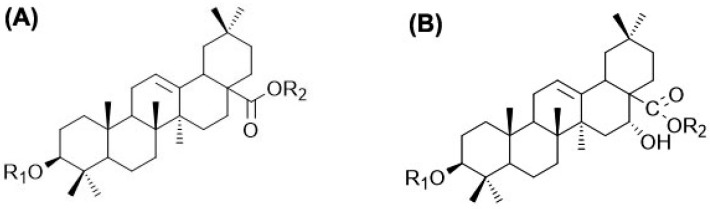
Chemical structures of triterpene glycosides Araloside V, 4-F8 (**A**), and Araloside VI (**B**). Adapted from a study by Xia et al. [86].

The lengths of the sampled buds were measured, and the buds were dried in an oven at 70 °C for 72 h. The dried samples were crushed into powders, and 100 mg portions were immersed in 2 mL of 70% methanol–water solution. The mixture was ultrasonicated for 30 min at 80 °C and 28 kHz. The solution was then centrifuged at 12,000 rpm for 10 min at 4 °C. The supernatant was collected and filtered through a 0.22 µm syringe filter prior to a mass spectrometry analysis. The TGs of Araloside V, Araloside VI, and 4-F8 were determined in terms of their activated responses to environmental changes [39,85,86] (Figure 9). Araloside V (R_1_ = Glc (Glc)_3_-2,3 (Glc), R_2_ = Glc) and 4-F8 (R_1_ = Glc-3 (Glc)-6 (Glc), R_2_ = Glc) structural analogs share the same core structure and R_2_ group but differ in the glycosidic substitution pattern at R_1_, which makes them part of the same family of compounds but with distinct sugar arrangements (Figure 9A). The core structure of Araloside VI (R_1_ = Glc (Glc-2,3) Glc, R_2_ = Glc) features a polycyclic ring system with hydroxyl groups (Figure 9B), suggesting that the complex glycosylated compound is completely different from those in Araloside V and 4-F8.

A generic strategy was employed, based on the gas-phase decomposition of protonated and ammoniated precursors (DPAP) coupled with ultra-performance liquid chromatography–multiple reaction monitoring–mass spectrometry (UPLC-MRM-M), which has been proven to efficiently determine these three compounds. HPLC-grade formic acid and acetonitrile were used, along with deionized water from a Milli-Q purification system. Araloside V, Araloside VI, and 4-F8 reference standards were either purchased or isolated with purities exceeding 97%, as determined with an LC-MS analysis. HPLC-grade formic acid and acetonitrile were used along with deionized water from a Milli-Q purification system. Reference standards were purchased from a biochemical agency (Must BioTech Inc., Chengdu, China) with purities exceeding 97%, as required for the LC-MS analysis. Individual stock solutions of each standard (1 mg mL ^−1^) were prepared by accurately weighing and dissolving them in HPLC-grade methanol. The stock solutions were then mixed with methanol to obtain a final mixed standard solution (10 µg mL ^−1^).

A Waters ACQUITY UPLC H-Class system (Waters Inc., Milford, MA, USA) featuring an HSS T3 column (2.1 × 150 mm, 1.8 µm) and a Waters HSS T3 guard column was used for the liquid chromatography. The column temperature was maintained at 35 °C in a sample room at 10 °C. Mobile phase A consisted of 0.1% formic acid in water, and mobile phase B consisted of 0.1% formic acid in acetonitrile. The UPLC gradient for the bud samples involved a multistep elution program. Mass spectrometry was conducted on a 4000 Qtrap mass spectrometer (SCIEX Corp., Framingham, MA, USA) with a TurboIonspray interface operating in the positive ion mode. The ion spray voltage was set at 5500 V, the turbo spray temperature was 300 °C, and the interface heater was turned on. The nebulizer and heater gases (nitrogen) were set at 50 psi. The curtain gas, entrance potential, and cell exit potential were set as 25, 10, and 10, respectively. The injection volumes for both standards and samples were 2 µL. A central composition design (CCD) using Design-Expert 8.6 software (Stat-Ease, Inc., Minneapolis, MN, USA) was used to investigate the effects of collision energy (CE) and declustering potential (DP) on the Multiple-Reaction-Monitoring (MRM) peak area. A partial least-squares regression (PLSR) analysis was performed using EZinfo Masslynx ver. 4.1 (Waters Corporation, Milford, MA, USA). The results were assigned as the percentage of triterpene amounts relative to the dry weight mass of the buds.

### 4.6. Variable Calculation and Statistics

The LSF impact was evaluated using different variables for cumulative days above 0 °C (CD0) [5,87], 12 °C (CD20) [74,75], and 20 °C (CD20) [6,68,75]. The number of LSF (NLSF) and days of LSF (DLSF) are shown in Figure 8. The days of temperature rise (DTR) determine the time range of warm spells at an earlier stage, which further determines the bud burst in spring [2,68]. The days of temperature decline (DTDs) after warm spells have been proven to have a species-specific effect in reducing the relative conductance of bud cells, suggesting impairment of the membrane through freezing damage [75,88]. The highest temperature in the LSF (HTLSF) showed contrasting impacts on vulnerability to LSF [20,89]; however, evidence on *A. elata* is not clear. The degree of harm inflicted during a frost event depends on the lowest temperature encountered (the lowest temperature during temperature decline, LTLSF), as subfreezing conditions induce freezing injury and may lead to the demise of plant tissues [90]. The rate of temperature change, particularly warming trends, influences the frequency and severity of these events and the timing of spring phenology [8,9,68]. This can be calculated as follows:

(1) $$TCR=\frac{T_{1}-T_{0}}{D_{LSF}}$$

where *TCR* is the temperature change rate, *T*_0_ and *T*_1_ are temperatures at the start of the year and the day of bud sampling, respectively, and *D_LSF_* is the number of days of LSF between *T*_1_ and *T*_0_. In the early stage, when a warm spell forms, *TCR* refers to the temperature rise rate (TRR); in the later stage, during a sudden temperature decline, *TCR* refers to the temperature decline rate (TDR).

The data were analyzed using R software version 4.1.2 (R Foundation for Statistical Computing, Vienna, Austria). The analysis of variance (ANOVA) was conducted to determine the effects of variations in the counties on the bud dry weight and bud length. The one-way ANOVA was conducted to reveal the effects of the forest type and provincial disparity on bud TGs, soil properties, and LSF attributes. When significant results were detected, they were compared using Duncan test at a level of 0.05. Pearson correlation was used to detect relationships among pairs of variables related to the soil properties or LSF attributes. Correlations were also used to detect the geographical responses of bud TGs to longitudinal or latitudinal gradients. Finally, multivariate linear regression was used to detect multiple facets across LSF attributes and soil properties contributing to the bud TGs.

## Figures and Tables

**Table 1:** Provincial differences in LSF attributes for *A. elata* populations located in counties between Heilongjiang and Jilin were disclosed with analysis of variance (ANOVA) results.

Variable	Province		ANOVA	
	Heilongjiang	Jilin	*F* Value	*p* Value
CD0 ^1^ (d)	67.8 ± 4.6 a	63.6 ± 5.9 b	4.60	0.0384
CD12 ^1^ (d)	26.4 ± 3.4 a	23.8 ± 3.6 a	3.88	0.0562
CD20 ^1^ (d)	6.0 ± 2.5 a	4.4 ± 04 b	4.24	0.0463
NLSF ^2^ (time)	1.8 ± 0.5 a	1.2 ± 0.4 b	12.03	0.0013
DLSF ^2^ (d)	21.5 ± 5.2 a	17.4 ± 3.9 b	5.51	0.0242
DTR ^3^ (d)	15.9 ± 3.8 a	12.0 ± 3.3 b	8.46	0.0060
DTD ^3^ (d)	5.6 ± 2.1 a	5.0 ± 1.0 a	0.84	0.3662
HTLSF ^4^ (°C)	20.5 ± 2.8 b	22.6 ± 0.4 a	5.97	0.0193
LTLSF ^4^ (°C)	−5.5 ± 2.0 a	−5.0 ± 1.9 a	0.47	0.4980
TRR ^5^ (∆°C d ^−1^)	1.9 ± 0.4 a	1.8 ± 0.3 a	0.94	0.3375
TDR ^5^ (∆°C d ^−1^)	−1.2 ± 0.4 a	−1.7 ± 0.3 b	14.84	0.0004

Note: ^1^ CD0, CD12, CD20, cumulative days with lowest temperature over 0 °C, 12 °C, and 20 °C, respectively; different letters indicate significant differences between the two provinces; ^2^ NLSF, DLSF, number and days of LSF, respectively; ^3^ DTR, DTD, days of temperature rise and decline, respectively; ^4^ HTLSF, LTLSF, highest and lowest temperatures in LSF, respectively; ^5^ TRR, TDR, temperature rise or decline rates, respectively.

**Table 2:** Summary of plot location information and forest types in *Aralia elata* populations.

Province	County	Latitude	Longitude	Forest Type
Heilongjiang	Boli	45.69°–45.71°	130.35°–130.55°	SF ^1^, PP ^2^
Heilongjiang	Hegang	47.40°–47.52°	130.14°–130.31°	LP ^3^, RPDBF ^4^
Heilongjiang	Hulin	45.88°–45.98°	132.39°–132.62°	BF ^5^, SF
Heilongjiang	Jiamusi	46.58°–47.00°	129.72°–130.63°	FP ^6^, LP, RPDBF
Heilongjiang	Mudanjiang	44.76°–44.89°	129.10°–129.26°	FP, BF, LP
Heilongjiang	Raohe	46.80°–46.95°	133.75°–133.90°	SF
Heilongjiang	Shangzhi	45.22°–45.40°	127.10°–127.26°	SF, PP
Heilongjiang	Shuangyashan	46.36°–46.53°	131.09°–131.20°	BF, SF, RPDBF
Heilongjiang	Wuying	48.05°–48.20°	129.17°–129.61°	FP
Heilongjiang	Yichun	47.48°–47.81°	128.95°–129.39°	BF, SF, LP
Jilin	Antu	42.17°–42.29°	128.14°–128.20°	SF, PP, SF
Jilin	Fusong	42.16°–42.23°	127.50°–127.52°	RPDBF
Jilin	Jiangyuan	41.92°–41.95°	126.52°–126.56°	LP
Jilin	Jingyu	42.43°–42.56°	126.76°–126.80°	SF, PP
Jilin	Linjiang	41.80°–41.86°	127.04°–127.05°	RPDBF, LP

Note: ^1^ SF, second forest; ^2^ PP, pine plantation; ^3^ LP, larch plantation; ^4^ RPDBF, red pine (*Pinus koraiensis*) and deciduous broadleaf mixed forest; ^5^ BF, birch forest; ^6^ FP, fir plantation.

**Table 3:** Specific identification names of the U.S. Air Force Stations (USAFS) were located at the closest distance from the host counties for *A. elata* plots with sampling dates.

Host County	USAFS Code	Sampling Date
Boli	509780	9 May
Hegang	507750	14 May
Hulin	509830	10 May
Jiamusi	508730	13 May
Mudanjiang	509630	5 May
Raohe	508880	8 May
Shangzhi	509530	28 April
Shuangyashan	509780	12 May
Wuying	507750	14 May
Yichun	507740	13 May
Antu	543860	3 May
Fusong	542840	28 April
Jiangyuan	543770	25 April
Jingyu	542730	21 April
Linjiang	543740	23 April

## Data Availability

The raw data supporting the conclusions of this article will be made available by the authors on request. The data are not publicly available due to the restrictions in grant.
